# Dynamic Monitoring of Cellular Remodeling Induced by the Transforming Growth Factor-β1

**DOI:** 10.1007/s12575-009-9017-9

**Published:** 2009-09-12

**Authors:** Andrea Staršíchová, Lukáš Kubala, Eva Lincová, Zuzana Pernicová, Alois Kozubík, Karel Souček

**Affiliations:** 1 Department of Cytokinetics, Institute of Biophysics, Academy of Sciences of the Czech Republic, Královopolská 135, 612 65, Brno, Czech Republic; 2 Department of Free Radical Pathophysiology, Institute of Biophysics, Academy of Sciences of the Czech Republic, Brno, Czech Republic

**Keywords:** real-time cell analysis, cell plasticity, epithelial–mesenchymal transition, transforming growth factor-β1, F-actin, cytoskeleton remodeling

## Abstract

The plasticity of differentiated adult cells could have a great therapeutic potential, but at the same time, it is characteristic of progression of serious pathological states such as cancer and fibrosis. In this study, we report on the application of a real-time noninvasive system for dynamic monitoring of cellular plasticity. Analysis of the cell impedance profile recorded as cell index using a real-time cell analyzer revealed its significant increase after the treatment of prostate epithelial cells with the transforming growth factor-β1. Changes in the cell index profile were paralleled with cytoskeleton rebuilding and induction of epithelial–mesenchymal transition and negatively correlated with cell proliferation. This novel application of such approach demonstrated a great potential of the impedance-based system for noninvasive and real-time monitoring of cellular fate.

## 1. Introduction

The phenomenon of plasticity of differentiated adult cells could have a great therapeutic potential, but at the same time, it is characteristic of progression of serious pathological states. Epithelial–mesenchymal transition (EMT) is a crucial process in embryogenesis, but it also occurs during progression of tumors derived from epithelial cells (for review, *see *[1]). The transforming growth factor-β1 (TGF-β1) is an important growth factor inducing remodeling of epithelial cells. TGF-β1 induces a complex change of the gene expression profile, which leads to the induction of cell cycle arrest, increased cell migration, and spreading [2-4]. In general, determination of the quality and quantity of remodeling of epithelial cells is a complex issue. It usually includes quantification of expression of epithelial and mesenchymal markers (E-cadherin, N-cadherin, and vimentin), visualization of cytoskeletal rebuilding (F-actin), migration, and invasive assay (wound healing and migration through Matrigel matrix; [5]). Conventionally, most of the approaches mentioned are based on a time-consuming end-point analysis of the state of whole cell populations combined with advanced techniques of analysis of individual cells with the use of flow cytometry or digital microscopic techniques and image analysis. However, neither the episodic nor the spatial resolution of these techniques is capable of registering very small and fast changes in cellular morphology. Currently, label-free and noninvasive methods based on electronic cell sensor arrays were suggested for the monitoring of cell physiology, particularly adhesion, spreading, and transient changes in cell morphology [6-9]. To widely accept this methodological approach and to correctly and precisely interpret data for these measurements is crucial to obtain precise correlation with cell morphology and overall phenotype using a relevant reference method. However, well-described models applying this methodological approach with different cell lines and various cell plasticity modulating conditions are missing. Here, we showed that the impedance-based real-time cell analyzer (RTCA) allows dynamic monitoring and quantification of cell remodeling during TGF-β1-induced EMT in non-transformed prostate epithelial cells. This novel application of such approach demonstrated a great medium-throughput potential of the impedance-based system for noninvasive and real-time monitoring of cellular fate.

## 2. Materials and methods

### 2.1. Cells

BPH-1 cells were obtained from the German Collection of Microorganisms and Cell Cultures and cultivated in RPMI 1640, supplemented with 20% bovine fetal serum (both PAA), 5 μg/ml transferrin, 5 ng/ml sodium selenite, and 5 μg/ml insulin (Invitrogen). The cell lines were cultivated in Nunc (Thermo Fisher Scientific) cultivation dishes, flasks, and plates in a humidified incubator at 37°C in an atmosphere of 5% CO_2_.

### 2.2. Real-time cell impedance analysis

Acea E-plates^®^ 96 were used for noninvasive real-time measurement with the use of an xCELLigence RTCA SP system including RTCA Software version 1.1 (both Roche). First, a standard background measurement was performed using 100 μl of complete cultivation media. BPH-1 cells were trypsinized, quantified, and seeded in additional 100 μl of cultivation media in a final concentration of 30,000 cells per cm^2^. The cells were monitored continually every 1 min in the first 45 min after the seeding and then every 1 h for a period of 96 h. Recombinant TGF-β1 (Millipore) treatment with various concentrations in triplicate was performed 24 h after the seeding of the cells. Formation of contractile microfilaments was blocked by cytochalasin B (CB), *Helminthosporium dematioideum* (Calbiochem) dissolved in methanol (MeOH). The cells were pretreated with TGF-β1 (10 ng/ml) for 68 h and treated with CB (10 μg/ml) for another 3 h. The cells were monitored continually every 15 s after the CB addition. In this case, data are presented as a normalized cell index (CI; normalized at the time of 68 h). Cultivation of the cells and their treatment were performed under standard conditions (37°C/5% CO_2_).

### 2.3. Cell counts

The numbers of trypsinized BPH-1 cells in the culture were determined using a Coulter Counter^®^ ZM (Beckman-Coulter).

### 2.4. ATP assay

Intracellular ATP was detected in BPH-1 cells by the commercial ATP cellular kit (Biothema, Sweden). The cells were incubated according to the experimental procedure, the supernatant was removed, and the cells were lysed by the Somatic cell ATP releasing reagent (Sigma-Aldrich). Then, 50 μl of lysate was mixed with 20 μl of ATP reagent containing D-luciferin, luciferase, and stabilizers. Intracellular ATP contents were determined using a microplate luminometer LM-01T (Immunotech).

### 2.5. Fluorescent and light microscopy

F-actin was visualized after the staining of paraformaldehyde (2%) fixed and permeabilized BPH-1 cells with phalloidin-fluorescein isothiocyanate (Sigma-Aldrich) using a fluorescent microscope (Olympus IX-70, Fluoview II CCD camera). Nuclear counterstaining was performed by using 4',6-diamidine-2'-phenylindole dihydrochloride (DAPI; Fluka). Cell morphology was documented by phase contrast on the same microscope.

### 2.6. Western blot

BPH-1 cells were treated by various concentrations of TGF-β1 for different time intervals and harvested in radioimmunoprecipitation assay buffer (50 mM Tris–HCl pH 7.4, 1% NP-40, 0.25% sodium deoxycholate, 150 mM NaCl, protease inhibitor cocktail, and phosphatase inhibitor cocktail set II (Merck)). Protein concentration was determined using detergent-compatible protein assay (Bio-Rad). The cell lysates were sonicated (5 s, Sonifier^®^ B-12, Branson Ultrasonics Corp), spun, and mixed with 3× sodium dodecyl sulfate (SDS) loading buffer (240 mM Tris–HCl pH 6.8, 6% SDS, 0.02% bromphenol blue, 30% glycerol, 3% β-mercaptoethanol). Equivalent quantities of protein (20 μg) were separated by SDS-polyacrylamide gel electrophoresis and transferred onto polyvinylidene fluoride membranes (Millipore) using established procedures. The membranes were blocked in Tris-buffer saline (20 mM Tris–HCl pH 7.2, 140 mM NaCl) containing 0.1% Tween 20 and 5% non-fat milk. The levels of phosphorylated (Ser465/467) and total Smad2, and expression of vimentin, a characteristic mesenchymal marker, were analyzed with specific primary antibodies (Cell Signaling and Sigma-Aldrich). Anti-β-actin (A5441) was from Sigma-Aldrich; horseradish peroxidase-conjugated anti-mouse IgG (#NA931) and anti-rabbit IgG (#NA934) were from GE Healthcare. Detection of antibody reactivity was performed using Immobilon Western HRP Substrate (Millipore). Densitometric measurements were performed using ImageJ software (NIH) and normalized to the expression of β-actin.

## 3. Results and discussion

**Figure 1:**
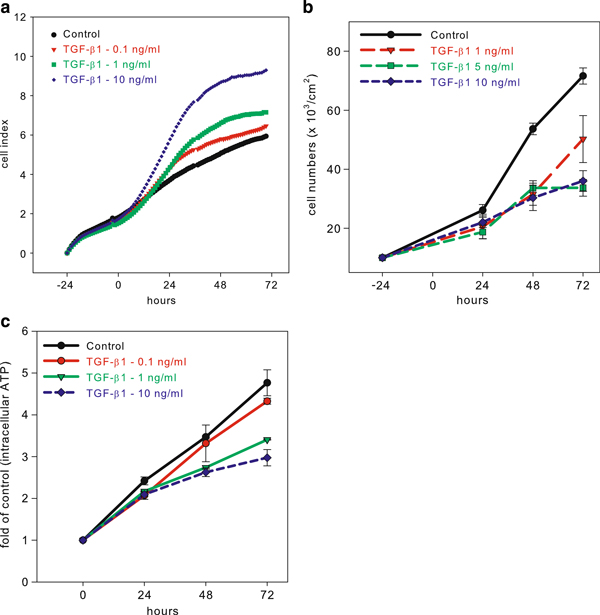
**The antiproliferative effect of transforming growth factor-β1 (TGF-β1) is associated with the increase of the cell index (impedance) in BPH-1 cells**. **a** TGF-β1 induces an increase of the cell index (impedance) acquired with the use of an xCELLigence real-time cell analyzer system in a time- and concentration-dependent manner. However, at the same time, TGF-β1 strongly inhibits cell proliferation quantified by counting of the cells using a Coulter Counter (**b**), and by ATP assay (**c**). The cells were treated with various concentrations of TGF-β1 at the time marked as time zero.

Data acquisition demonstrated a linear increasing of the CI values in control cells during the time interval observed. However, this linear trend was significantly changed by TGF-β1 in less than 12 h after the treatment in a concentration-dependent manner (Figure 1a). Concentrations of 1 and 10 ng/ml induced a significant steep increase in CI values, which reached a plateau in 48 h. The impedance-based determination is by its nature dependent on the number of adherent cells. Thus, we compared CI determination with the analysis of cell numbers to clarify the contribution of changes in cell numbers and the morphological alternation of cells to detected CI values. In parallel with the E-plates^®^ 96, the cells were seeded at the same density on 40 mm dishes and 4-well plates, and treated with TGF-β1 in the same experimental design. At various time intervals after the treatment, the numbers of trypsinized cells were determined using a Coulter Counter simultaneously with the determination of metabolically active viable cells based on determination of intracellular ATP in cell lysate. Our data, shown in Figure 1b, c, demonstrate that TGF-β1 induced antiproliferative effects in BPH-1 cells in a time- and concentration-dependent manner. These trends are in negative correlation with CI values acquired with the use of RTCA. Taken together, these data showed that the TGF-β1 induced antiproliferative effects in BPH-1 cells are paralleled by an increase of cell impedance.

**Figure 2:**
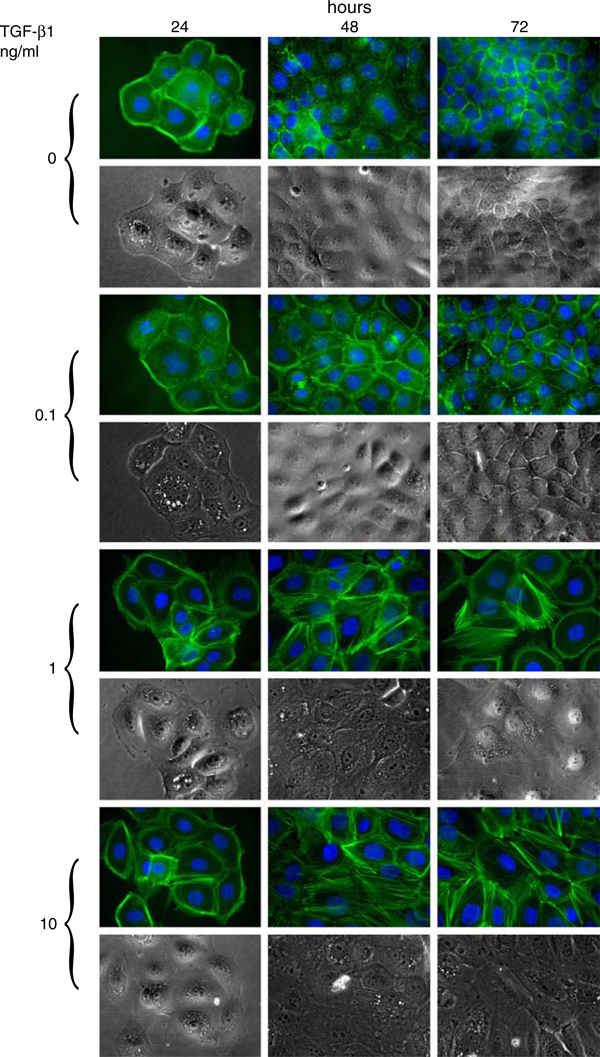
**The effect of transforming growth factor-β1 in BPH-1 cells is associated with remodeling of the cytoskeleton**. F-actin was visualized with the use of phalloidin-fluorescein isothiocyanate conjugate. The nuclei were counterstained with DAPI. Images of cellular morphology were taken under phase contrast.

**Figure 3:**
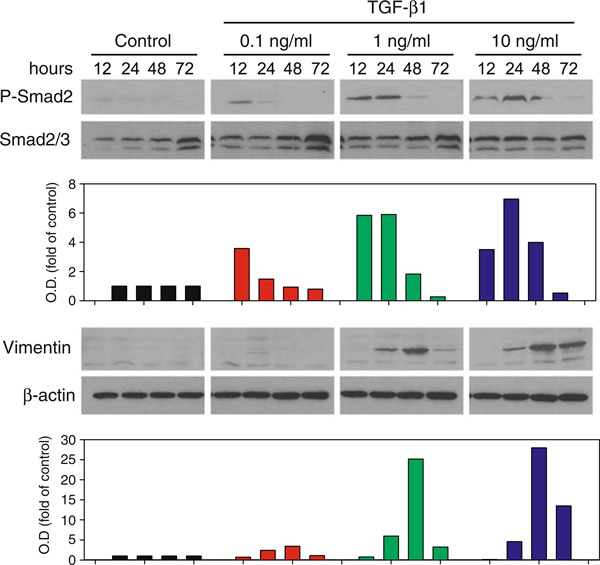
**Transforming growth factor-β1 induces phosphorylation of Smad2 and epithelial–mesenchymal transition in BPH-1 cells**. The cells were lysed using radioimmunoprecipitation assay buffer, sodium dodecyl sulfate-polyacrylamide gel electrophoresis, and immunoblotting detection of p-Smad2 (Ser465/467); Smad2 and vimentin was performed. Detection of β-actin served as control of equal loading. Densitometric measurements were performed using ImageJ software (NIH, Bethesda, MD, USA) and normalized to the expression of β-actin. The *bar graph* represents the average optical density.

**Figure 4:**
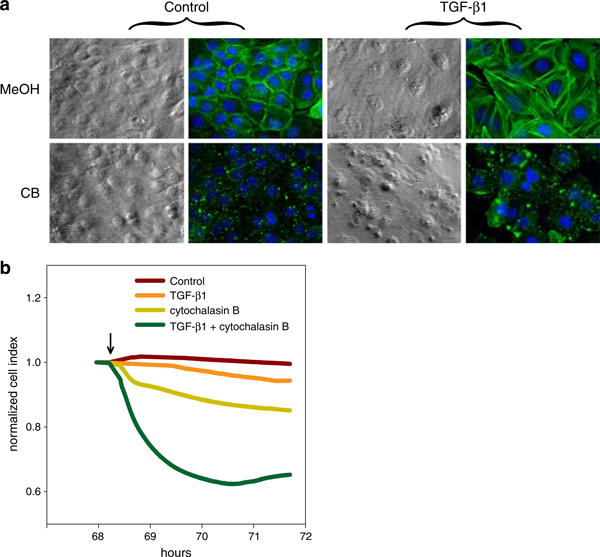
**Inhibition of formation of contractile microfilaments by cytochalasin B (CB) leads to a rapid drop of cell index values**. BPH-1 cells were pretreated with transforming growth factor-β1 (10 ng/ml) for 68 h and treated with CB (10 μg/ml) for another 3 h. **a** F-actin was visualized by phalloidin-fluorescein isothiocyanate conjugate after 3 h of CB and/or vehicle treatment. **b** CB induces a rapid decrease of the normalized cell index (data were normalized at the time of 68 h) acquired with the use of an xCELLigence real-time cell analyzer system. The *arrow* indicates the time interval of CB addition.

It is a well-known fact that when stimulated with TGF-β1, epithelial cells undergo EMT and exhibit a significant formation of actin stress fibers that emanate from focal adhesions [10]. In this case, our results demonstrate the usual uncertainty of image analysis which is limited by confluence of the cells. The control cells reached a relatively high density after 72 h of cultivation. Based on the F-action visualization and cell morphology analysis, there is no significant difference between control cells and cells treated with 0.1 ng/ml of TGF-β1. However, it is evident that TGF-β1 at 1 and 10 ng/ml concentrations induced formation of F-actin stress fibers and increased cell spreading (Figure 2). These results positively correlate with the activation of Smad-dependent signaling and EMT by TGF-β1 (Figure 3). Our results showed transient phosphorylation of Smad2 induced by both 1 and 10 ng/ml of TGF-β1, which is followed by massive induction of vimentin expression. TGF-β1 at a concentration of 0.1 ng/ml did not induce vimentin expression and only slightly induced phosphorylation of Smad2. Furthermore, we wanted to examine the influence of formation of stress fibers on TGF-β1-induced increase of CI values. CB, a potent inhibitor of the formation of contractile microfilaments, induced inhibition of F-actin formation in both control and TGF-β1-treated BPH-1 cells (Figure 4a). This inhibition was paralleled by a rapid decrease of normalized CI values in both groups (Figure 4b). However, the drop of CI values induced by CB was much stronger in the case of TGF-β1-pretreated BPH-1 cells, with high abundance of F-actin stress fibers in comparison with CB-treated control cells. These results demonstrate that TGF-β1-induced increase of CI values is at least partially dependent on stress fiber formation. We can summarize that TGF-β1-induced EMT in BPH-1 cells is associated with inhibition of proliferation, rebuilding of cytoskeleton, formation of stress fibers, and increase of spreading. These cellular changes are associated with a significant increase in CI values analyzed by the RTCA system. Until now, most of the applications of RTCA focused on analysis of adhesion or spreading were designed to study immediate interaction of cells with substrate in relatively short time intervals [6,11]. Here, our data suggest a novel application and a great potential of real-time impedance measurement for dynamic monitoring of cellular remodeling and plasticity, induced after adhesion of the cells to the substrate and formation of stress fibers in longer time intervals. Furthermore, these measurements also show a potential misinterpretation of the data in experimental setups using impedance-based methods for the detection of cell proliferation without simultaneous monitoring of cellular morphological changes.
